# Decidual γδT cells of early human pregnancy produce angiogenic and immunomodulatory proteins while also possessing cytotoxic potential

**DOI:** 10.3389/fimmu.2024.1382424

**Published:** 2024-03-27

**Authors:** Jasper Nörenberg, Péter Vida, Isabell Bösmeier, Barbara Forró, Anna Nörenberg, Ágnes Buda, Diana Simon, Szabina Erdő-Bonyár, Pál Jáksó, Kálmán Kovács, Éva Mikó, Tímea Berki, Emese Mezősi, Alíz Barakonyi

**Affiliations:** ^1^ Department of Medical Microbiology and Immunology, University of Pécs Medical School, Clinical Center, Pécs, Hungary; ^2^ National Laboratory on Human Reproduction, University of Pécs, Pécs, Hungary; ^3^ Department of Obstetrics and Gynaecology, University of Pécs Medical School, Clinical Center, Pécs, Hungary; ^4^ Department of Pathology, University of Pécs Medical School, Clinical Center, Pécs, Hungary; ^5^ Janos Szentagothai Research Centre, University of Pécs, Pécs, Hungary; ^6^ Department of Immunology and Biotechnology, University of Pécs Medical School, Clinical Center, Pécs, Hungary; ^7^ First Department of Internal Medicine, University of Pécs Medical School, Clinical Center, Pécs, Hungary

**Keywords:** decidua, γδT cells, HLA-E, HLA-G, NK receptors, cytokines, angiogenic factors, cytotoxic mediators

## Abstract

During pregnancy, the maternal immune system must allow and support the growth of the developing placenta while maintaining the integrity of the mother’s body. The trophoblast’s unique HLA signature is a key factor in this physiological process. This study focuses on decidual γδT cell populations and examines their expression of receptors that bind to non-classical HLA molecules, HLA-E and HLA-G. We demonstrate that decidual γδT cell subsets, including Vδ1, Vδ2, and double-negative (DN) Vδ1-/Vδ2- cells express HLA-specific regulatory receptors, such as NKG2C, NKG2A, ILT2, and KIR2DL4, each with varying dominance. Furthermore, decidual γδT cells produce cytokines (G-CSF, FGF2) and cytotoxic mediators (Granulysin, IFN-γ), suggesting functions in placental growth and pathogen defense. However, these processes seem to be controlled by factors other than trophoblast-derived non-classical HLA molecules. These findings indicate that decidual γδT cells have the potential to actively contribute to the maintenance of healthy human pregnancy.

## 1 Introduction

During pregnancy, the coexistence of two genetically and immunologically different individuals within one body challenges primary transplantation and tumor physiology concepts. In their context, the maternal immune system’s task is to ensure the integrity of the mother’s body and remove foreign or dysplastic tissues. However, the maternal immune system does not attack embryonal or fetal tissues but supports implantation, placentation, and fetal growth (1–3).

In human pregnancy, the trophoblast infiltrates deeply into the decidua and spiral arteries, allowing the establishment of a hemochorial placenta. This type of placentation, in which maternal blood is in direct contact with fetal tissues, ensures a sufficient supply of oxygen and nutrients. Insufficient supply, caused by weak trophoblast invasion during the first trimester, may lead to human pregnancy disorders, like fetal growth restriction or preeclampsia (4–6). Furthermore, early pregnancy loss or infertility cases might be connected to even weaker implantation and invasion. Research of the last decades emphasizes the importance of the interaction of trophoblast and decidua for controlling invasion depth and establishing a healthy placenta (7–9). Cases of the placenta accreta spectrum, in which the trophoblast might even invade neighboring organs, highlight the role of the decidua in this process as they commonly occur when the blastocyte implants at the site of a uterine scar, where decidua is absent (9–11).

**Figure 1:**
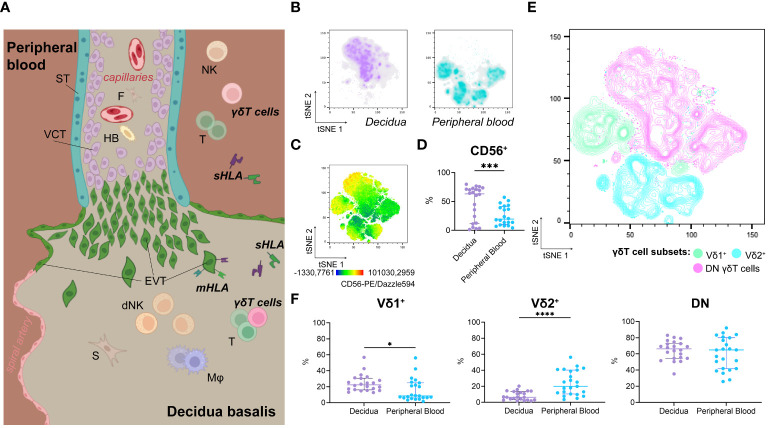
**(A)** Illustration of anatomical structure and spatial relations at the feto-maternal interface. **(B)** Representative isolated depiction of tSNE-clustered decidual (left) and maternal peripheral blood (right) γδT cells as density plots (n=1; Panel A, also see **Supplementary Information 1B**). **(C)** Representative tSNE-plot depicting fluorescence intensity of CD56-PE/Dazzle594™ on γδT cells from peripheral blood and decidua (n=1; Panel A, also see **Supplementary Information 1C**). **(D)** Statistical comparison of CD56^+^ cells’ prevalence among γδT cells from the decidua (n=22) and peripheral blood (n=23) (see also **Supplementary Information 1D**). **(E)** Representative contour plot overlay of Vδ1^+^, Vδ2^+^, and DN γδT cells on clustered γδT cells from peripheral blood and decidua (n=1; Panel A, also see **Supplementary Information 1E**). **(F)** Statistical comparison of Vδ1^+^, Vδ2^+^, and DN γδT cells’ prevalence among γδT cells from the decidua (n=22) and peripheral blood (n=23). Testing for significance was performed with the Wilcoxon test. *: p ≤ 0.05, ***: p ≤ 0.001, ****: p ≤ 0.0001; ST, syncytiotrophoblast; VCT, villous cytotrophoblast; F, fibroblast; HB, Hofbauer cell; (d)NK, (decidual) Natural killer cell; T, T cell; s/mHLA, soluble/membrane-bound Human Leukocyte Antigen class I; EVT, extravillous cytotrophoblast; S, stroma cell; Mφ, Macrophage.

At the time of receptivity (window of implantation), leukocytes accumulate in the decidua, dominated by a unique CD56^bright^ innate lymphoid cell population, commonly known as uterine/decidual NK (u/dNK) cells (12). Decidual NK cells have an array of activating and inhibiting receptors, which bind specific classical and non-classical HLA class I molecules (13–17). The extravillous cytotrophoblast (EVT), which is in direct contact with the decidua and invades uterine spiral arteries (**Figure 1A**), is unique in its HLA class I expression pattern: The highly variable HLA-A and -B are not expressed by the EVT. Instead, its cells express HLA-C and the oligomorphic HLA-E and -G on their cell surface. Under physiological circumstances, HLA-G is exclusively known to be expressed by the EVT (18, 19). Next to the membrane-bound form of HLA-E and HLA-G (mHLA-E/-G), soluble forms (sHLA-E/-G) have been found in the sera of pregnant women (20–23).

Next to dNK cells, γδT cells and their potential roles during pregnancy have attracted interest. They are well known to surveil the tissue integrity of frontiers between the organism and the environment (24, 25). Studies reported a higher prevalence of γδT cells among decidual CD3^+^ cells compared to the peripheral blood (26–28). These decidual γδT cells, like dNK cells, are either clustered proximate to decidual glands or scattered as intraepithelial lymphocytes (26). The association of decidual γδT cells and dNK cells to glands might be connected to the invasion of the EVT, as the EVT penetrates not only spiral arteries but uterine glands (29). A growing body of evidence attributes a central role to these glands, providing nutrients, growth factors, and cytokines during placentation (29–31).

Innate lymphoid cells, like dNK cells, and γδT are closely related. Although γδT cells have an antigen-recognition receptor, they rely on an arsenal of cytotoxicity receptors for their activity. Furthermore, in contrast to αβT cells, they are not MHC-restricted for their antigen recognition. However, some of these cytotoxicity-related receptors bind HLA class I molecules and transmit activating or inhibiting signaling upon ligation. Consequently, HLA expression is likely to influence γδT cell behavior.

Considering the EVT’s unique HLA class I expression profile, three receptor groups come into focus at the maternal-fetal interface. The NKG2 receptor family, with a particular emphasis on activating NKG2C and inhibitory NKG2A, plays a pivotal role in recognizing HLA-E expression (32, 33). HLA-G, conversely, can be bound by KIR2DL4, a receptor from the Killer cell Immunoglobulin-like Receptor (KIR) family (CD158). This family is mainly known for HLA-C binding. However, KIR2DL4 stands out among the KIR receptor family due to its unique ligand preference, location, and function. Unlike other KIR receptors, KIR2DL4 is predominantly located intracellularly and is only expressed on the cell surface during activation states. Although its molecular structure suggests an inhibitory function, KIR2DL4-ligation was shown to trigger cytokine release (34–36). Last, Immunoglobulin-like transcript (ILT2), a member of the leukocyte immunoglobulin-like receptor subfamily B, binds HLA class I molecules, including HLA-G, and transmits an inhibitory signal upon ligation (17, 33).

In addition, the non-classical MHC molecule CD1d, which presents lipid antigens, has the potential to facilitate antigen recognition through the γδTCR (37–39). However, further signals will influence the crosstalk between maternal γδT cells and the fetal EVT in the decidua. Although, investigations in tumor immunology have already demonstrated the expression of different non-classical HLA receptors (40), a detailed expression profile of these receptors on decidual γδT cells has not been published.

Here, we present an expression profile for HLA-E- or HLA-G-binding receptors of decidual γδT cells during early pregnancy. Furthermore, we investigated the potential consequences of respective receptor-ligand interactions. In this context, we focused on γδT cells’ secretion of mediators, which may influence vascular transformation or pathogen defense.

## 2 Results

### 2.1 Heterogeneity of peripheral and decidual γδT cells during early pregnancy

We found no significant difference between decidual and peripheral blood γδT cells’ prevalence among CD45^+^/live cells (**Supplementary Information 1A**). To characterize decidual γδT cells and compare them to their circulating counterparts, we utilized the downsampling plugin of FlowJo™ and concatenated previously gated γδT cell populations from decidual mononuclear cells (DMCs) and peripheral blood mononuclear cells (PBMCs). Defined separate clusters with minimal overlap were assigned to decidual or peripheral blood γδT cells, respectively (**Figures 1A, B**, see also **Supplementary Information 1B**).

Due to the biological similarities between NK cells and γδT, we investigated the expression of CD56 on γδT cells. While CD56^dim^ expression was detectable in several peripheral blood γδT cell clusters, decidual γδT cells exhibited both CD56^dim^ and CD56^bright^ phenotypes (**Figure 1C**, see also **Supplementary Information 1C**). Nevertheless, CD56^+^ γδT cells are more prevalent in the decidua than in the periphery (**Figure 1D**, see also **Supplementary Information 1D**).

Classical γδT cell subsets were associated with distinct clusters. Vδ1^+^ (CD45^+^TCRγδ^+^Vδ1^+^Vδ2^–^) cells were more prevalent in the decidua, while Vδ2^+^ (CD45^+^TCRγδ^+^Vδ1^–^Vδ2^+^) cells were more common among circulating γδT cells. However, double-negative (DN, CD45^+^TCRγδ^+^Vδ1^–^Vδ2^–^) γδT cells were the most common in both decidual and peripheral blood (**Figures 1E, F**, see also **Supplementary Information 1F**).

### 2.2 Decidual γδT subsets express receptors that bind to HLA-E or HLA-G molecules

**Figure 2:**
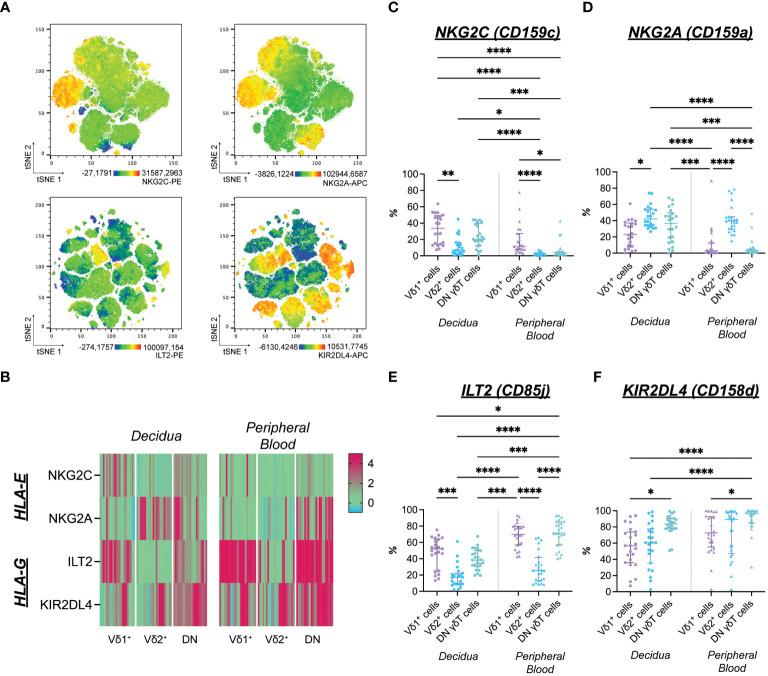
**(A)** Expression distribution of NKG2C-PE (upper left), NKG2A-APC (upper right) of flow cytometric Panel A, ILT2-PE (lower left, see also **Supplementary Information A/D**), and KIR2DL4-APC (lower right, see also **Supplementary Information A/D**) of flow cytometric panel B depicted as representative tSNE plots of paired, concatenated decidual and peripheral blood γδT cells (n=1). **(B)** Heatmap of standardized median fluorescence intensity ([Median_Subset_-Median_FMO_]/rSD_FMO_) of NKG2C-PE, NKG2A-APC, ILT2-PE, KIR2DL4-APC on matched decidual and peripheral blood γδT cell subsets (n=22; also see **Supplementary Information 2**). **(C)** Statistical comparison of NKG2C^+^ cells’ prevalence among γδT cells from the decidua (n=22) and peripheral blood (n=23). **(D)** Statistical comparison of NKG2A^+^ cells’ prevalence among γδT cells from the decidua (n=22) and peripheral blood (n=23). **(E)** Statistical comparison of ILT2^+^ cells’ prevalence among γδT cells from the decidua (n=22) and peripheral blood (n=23). **(F)** Statistical comparison of KIR2DL4^+^ cells’ prevalence among γδT cells from the decidua (n=22) and peripheral blood (n=23). Testing for significance was performed with the Kruskal-Wallis test. *: p ≤ 0.05, **: p ≤ 0.01, ***: p ≤ 0.001****: p ≤ 0.0001.

Using two flow cytometric panels (**Supplementary Information 6**), we investigated the prevalence and expression of HLA-E and HLA-G-binding receptors (NKG2C, NKG2A, and ILT2, KIR2DL4, respectively) on γδT cell subsets in the decidua and the matched peripheral blood (**Figure 2A**, see also **Supplementary Information 1B, E**). To estimate the expression intensity, we compared the median fluorescence intensity (normalized to the respective FMO) of all investigated receptors. Peripheral γδT cells show higher expression intensity for KIR2DL4 or ILT2 receptors than that for NKG2C and NKG2A. Within the different decidual γδT cell subpopulations, decidual DN γδT cells exhibited relatively high expression levels for all investigated receptors, while decidual Vδ1^+^ cells showed a more focused expression of the activating NKG2C and the inhibiting ILT2. In contrast, decidual Vδ2^+^ cells expressed significantly more NKG2A on their cell surface (**Figure 2B**, see also **Supplementary Information 2A, B**).

The prevalence of NKG2C^+^ cells was generally higher among decidual γδT cells compared to the periphery. However, this difference reached the level of significance only in the Vδ2^+^ and DN subsets. Furthermore, NKG2C positivity was significantly more common in the Vδ1^+^ subset compared to the Vδ2^+^ one (**Figure 2C**). Likewise, cells expressing the inhibitory counterpart NKG2A were more prevalent in the decidua. While the percentage of NKG2A^+^ cells among Vδ2^+^ cells did not differ between decidua and peripheral blood, a significantly higher proportion of DN γδT cells expresses NKG2A and NKG2C in the decidua compared to the periphery (**Figure 2D**).

The inhibitory HLA-G-binding ILT2 was commonly expressed by γδT cells independently of their origin. Generally, ILT2^+^ cells were less prevalent in the Vδ2^+^ subsets than in other γδT cell populations. However, when focusing on the prevalence of ILT2^+^ cells within each γδT cell subset, significantly fewer decidual DN γδT cells expressed ILT2 than their peripheral blood counterpart (**Figure 2E**). The HLA-G-binding KIR2DL4 was expressed by the majority of γδT cells (**Figure 2F**).

### 2.3 Decidual γδT cells secrete trophoblastotropic cytokines

**Figure 3:**
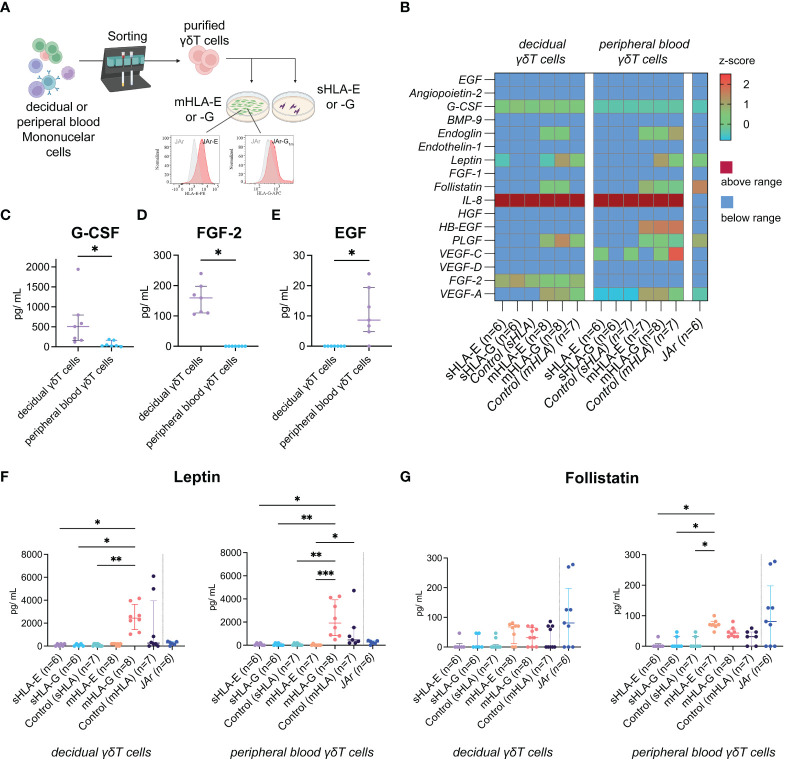
**(A)** Illustration of the experimental setup. **(B)** Heatmap depicting the z-score of the respective angiogenic factor after incubating peripheral blood (left) or decidual (right) γδT cells with soluble (s) or membrane-bound (m)HLA-E or -G. The last column depicts measurements from the human choriocarcinoma cell line JAr without γδT cells as an additional control (also see **Supplementary Information 3**). **(C)** Statistical comparison of G-CSF concentrations secreted from decidual (n=7) or peripheral blood (n=7) γδT cells without HLA molecules. Testing for significance was performed with the Wilcoxon matched-pairs signed rank test. **(D)** Statistical comparison of FGF-2 concentrations secreted from decidual (n=7) or peripheral blood (n=7) γδT cells without HLA molecules. Testing for significance was performed with the Wilcoxon matched-pairs signed rank test. **(E)** Statistical comparison of EGF concentrations secreted from decidual (n=7) or peripheral blood (n=7) γδT cells without HLA molecules. Testing for significance was performed with the Wilcoxon matched-pairs signed rank test. **(F)** Statistical comparison of Leptin concentrations measured after incubating peripheral blood or decidual γδT cells with sHLA or mHLA molecules. Testing for significance was performed with the Kruskal-Wallis test. **(G)** Statistical comparison of Follistatin concentrations measured after incubating peripheral blood or decidual γδT cells with sHLA or mHLA molecules. Testing for significance was performed with the Kruskal-Wallis test. *: p ≤ 0.05, **: p ≤ 0.01, ***: p ≤ 0.001.

To determine the functional consequences of the HLA-E or -G recognition by γδT cells, we incubated purified γδT cells with soluble HLA-E or -G (sHLA-E/-G). Furthermore, we utilized human choriocarcinoma cell lines (JAr) transfected with HLA-E or HLA-G_1m_ to investigate more complex interactions of membrane-bound HLA-E or -G (mHLA-E/-G) (**Figure 3A**).

Vascular transformation by the trophoblast and the local immune environment is crucial to establishing a healthy placenta during early pregnancy. Therefore, we analyzed the collected cell co-culture supernatants for potential angiogenic cytokines (**Figure 3B**). When comparing peripheral blood to decidual γδT cells without HLA molecules (“Control (sHLA)” in **Figure 3B**), we found significantly higher levels of G-CSF produced by the decidual ones (**Figure 3C**). Furthermore, decidual γδT cells produced FGF-2, whereas no FGF-2 was detected in the wells of peripheral γδT cells (**Figure 3D**). On the other hand, peripheral blood γδT cells produce small amounts of EGF, which was not detected in the wells of decidual samples (**Figure 3E**). While the production of most measured cytokines was not influenced by the presence or absence of HLA-E or -G molecules in our experimental model, incubating mHLA-G with γδT cells, independently from their origin, increased the measured Leptin concentrations (**Figure 3F**). Additionally, we detected elevated concentrations of Follistatin when incubating peripheral blood γδT cells with mHLA-E. However, compared to all other co-culture wells, the level of significance was not reached (**Figure 3G**).

### 2.4 Decidual γδT cells are strong producers of cytotoxic mediators

**Figure 4:**
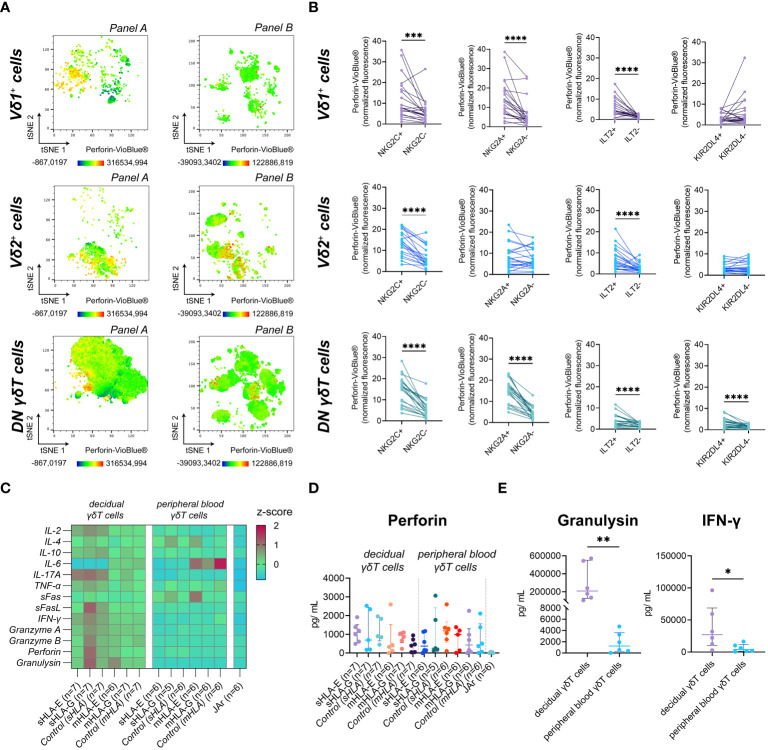
**(A)** Fluorescence intensity of Perforin-VioBlue^®^ on gated γδT cell subsets from the decidua for the two flow cytometric panels depicted as representative tSNE plots (n=1). **(B)** Statistical association of standardized Perforin-VioBlue^®^ median fluorescence intensity ([Median_Subset_-Median_FMO_]/rSD_FMO_) to the expression of NKG2C, NKG2A, ILT2, and KIR2DL4 on decidual γδT cell subsets (n=22). Testing for significance was performed with the Wilcoxon matched-pairs signed rank test. **(C)** Heatmap depicting the z-score of the respective soluble factor after incubating decidual (left) or peripheral blood (right) γδT cells with soluble (s) or membrane-bound (m) HLA-E or -G. **(D)** Statistical comparison of Perforin concentrations measured after incubating peripheral blood or decidual γδT cells with sHLA or mHLA molecules. Testing for significance was performed with the Kruskal-Wallis test. **(E)** Statistical comparison of Granulysin (left) and INF-γ (right) concentrations secreted from decidual (n=6) or peripheral blood (n=6) γδT cells in the absence of HLA molecules. Testing for significance was performed with the Wilcoxon matched-pairs signed rank test. *: p ≤ 0.05, **: p ≤ 0.01, ***: p ≤ 0.001, ****: p ≤ 0.0001.

γδT cells act as first responders in the mucosal defense against pathogens and many frontiers between the body and its environment. Therefore, we also analyzed the intracellular perforin content to determine each γδT cell subset’s cytotoxic potential in the decidua. Although in the periphery significantly less intracellular Perforin was measured in DN γδT cells compared to the Vδ2 population (**Supplementary Information 4A, B**), the Perforin content of the different γδT cell subsets did not differ in the decidua. **Figure 4A** confirms the distinct features of Perforin-positive decidual γδT cell subpopulations, as analyzed in our two flow cytometric antibody panels. (**Figure 4A**, see also **Supplementary Information 4A, B**).

Upon interaction with HLA-E or HLA-G, NKG2C, NKG2A, ILT2, and KIR2DL4 are potential regulators of the cytotoxic capability of immune cells. Investigating the perforin content of the different NK receptor-expressing decidual γδT cell populations, we found that the expression of NKG2C and ILT2 was associated with significantly higher levels of intracellular perforin in all decidual γδT cell subsets. The expression of NKG2A, however, correlated only in the Vδ1^+^ and DN γδT subset with higher levels of intracellular perforin. A similar, significant relation between KIR2DL4 expression and perforin content was only detectable in the DN γδT cell subset (**Figure 4B**).

To determine if this hypothetical relationship between cytotoxicity and the expression of HLA class I binding receptors has functional consequences, we analyzed the secretion of typical NK cell cytokines and cytotoxicity-related soluble molecules after exposure to sHLA-E/-G or mHLA-E/-G (**Figure 4C**, see also **Supplementary Information 4C-N**). However, the measured perforin concentration did not differ significantly (**Figure 4D**). In addition, we found that decidual γδT cells secrete excessive amounts of granulysin and high levels of interferon-γ (IFN-γ) (**Figure 4E**).

## 3 Discussion

Decidual γδT cells may have several functions in the microenvironment of the maternal-fetal interface between the decidua basalis and the extravillous trophoblast. They could influence the process of implantation and placentation via cytokine secretion or clear out pathogens.

The prevalence of γδT cells among CD45^+^/live cells was not different between the decidua and the peripheral blood. However, it is important to note that literature data shows that while the prevalence of T cells (CD3^+^) among all lymphocytes is around 70% in the peripheral blood, it is only around 15% in the early human decidua (41). Furthermore, according to previously published data, the population of γδT cells is markedly expanded in the decidua compared to the periphery (28, 41). Thus, due to the lack of CD3 staining, our prevalence data must be interpreted carefully.

As γδT cells exhibit innate and adaptive immunity aspects, they are often described as a bridge between these two parts of the immune system. Although a more didactic subdivision, γδT cells show quite some plasticity, and some subsets (e.g., Vγ9Vδ2) behave more in an innate manner, while others (e.g., Vδ1) show rather adaptive features (42–49). Therefore, when investigating γδT cells in any context, determining the prevalent subpopulations is a crucial first step before further conclusions.

The increase in the Vδ1 subset is typical for all tissues that frontier the environment and is often understood as a “first line defense” (24, 50). Our data on the prevalence of Vδ1 and Vδ2 subsets are in line with previously published flow cytometric data (51). However, these publications ignored the largest decidual γδT cell population, which expresses neither the Vδ1 nor the Vδ2 chain. Different variants of Vδ-chains exist, which makes DN γδT cells a heterogeneous population. However, a recent study used sequencing to show that the early human decidua is only inhabited by the Vδ1^+^, Vδ2^+^, and Vδ3^+^ γδT cell subsets (52). Therefore, DN γδT cells of the decidua can be considered as Vδ3^+^ cells. The Vδ3 subset is generally assumed to induce antigen-presenting cell maturation (53). In the context of pregnancy, the correct presentation of antigens is essential. Further investigations will be necessary to determine if decidual Vδ3 cells could direct antigen-presenting cell maturation toward a tolerance-promoting phenotype. Currently, anti-Vδ3 antibodies are not commercially available. Therefore, we cannot finally confirm that the DN subset represents the Vδ3-one, so henceforward, we refer to this population as DN γδT cells.

Interestingly, the majority of decidual γδT cells are CD56^+^, suggesting a special characteristic for decidual γδT cells. In addition, all three γδT cell populations contain cell clusters of CD56-expressing cells, which are more prevalent in the decidua. Although these decidual ‘NKγδT-like’ cells may be associated with dNK cells, as the expression of CD56 on lymphoid cells serves rather as a phenotypical marker, conclusions must be made carefully. To date, investigations indicating physiological similarities are still outstanding.

As mentioned previously, the trophoblast’s unique expression pattern of HLA class I molecules is crucial in allowing temporal chimerism during pregnancy. HLA molecules are absent at the maternal-fetal interface between the circulating maternal blood and the fetal syncytiotrophoblast in the intervillous spaces, which may explain why circulating adaptive immune cells do not drive an immune response at this location. However, the decidua directly interacts with the extravillous trophoblast expressing HLA-C, HLA-E, and HLA-G. Under physiological circumstances, HLA-G is uniquely expressed at this interface and is believed to influence the maternal immune system to accept and support viviparity. The expression of HLA-E, on the other hand, is connected to the expression of other HLA class I molecules, as it is loaded with their leader sequence peptides. Therefore, its expression on the cell surface usually is proportional to a given cell’s HLA class I expression level (54, 55). In cases of viral infection or malignancies, the expression of HLA class I molecules may alter, which consequently will be reflected in the expression of HLA-E. This can be detected by NK or cytotoxic T cells via the activating receptor NKG2C or the inhibitory receptor NKG2A. However, tumors may utilize upregulated HLA-E expression or secretion as an escape mechanism. Therefore, the extravillous trophoblast’s HLA-E positivity might simply result from its HLA-G expression.

Control of γδT cell function is essential, where trophoblast-expressed non-classical HLA molecules could serve as potent mediators. Among all decidual γδT cell subsets, cells expressing receptors for HLA-E or HLA-G are prevalent, suggesting an efficient influence of these non-classical HLA molecules. Especially the decidual Vδ1^+^ subset shows high expression levels of NKG2C and co-expresses NKG2A with lower intensity. However, considering the higher affinity of NKG2A to HLA-E, this difference in surface expression may be irrelevant (32). Both ILT2 and KIR2DL4 have been shown to bind various ligands, including HLA-G (33, 34). Next to its physiological expression and secretion by the EVT during pregnancy, malignancies are known to express and secret HLA-G. In this context, its ITL2-mediated inhibitory effects on Vγ9Vδ2 cells are well known [reviewed in (56)], and similar effects can be expected in the decidual microenvironment. KIR2DL4 is a unique Killer cell immunoglobulin-like receptor family member, as it is reportedly expressed on the cell surface or intracellularly by activated or non-activated NK cells. According to our findings, both decidual and peripheral γδT cells only express KIR2DL4 intracellularly. Considering its location, interactions with sHLA-G are more likely. However, intercellular HLA-G transfer through mechanisms like trogocytosis, nanotube transfer, or exosome provides an alternative possibility for KIR2DL4-HLA-G interactions (57). Despite its molecular structure suggesting an inhibitory function, it has been shown that the consequence of its ligation depends on the context. Rajagopalan et al., for example, demonstrated the production of angiogenic factors by peripheral blood NK cells due to KIR2DL4 ligation (36, 58).

For deeper investigation of the role of HLA-E and HLA-G in the functional control of different γδT cell subpopulations in the decidua, we analyzed the proportions of HLA class I-binding receptor expressing decidual γδT subsets. Decidual Vδ2 cells showed a reduced potential for activation and an increased potential for inhibition via HLA-E. Therefore, we suppose that HLA-E could be responsible, among others, for the control of Vδ2 cell function in the placenta. Regarding HLA-G, our data suggest that this non-classical HLA molecule preferentially regulates not only decidual γδT cells but also peripheral Vδ1 and DN γδT cells in its soluble form. These effects are mediated through ILT2 and KIR2DL4 receptor functions. In addition, because of the high ratio of KIR2DL4 expressing peripheral Vδ2 cells, soluble HLA-G is potentially able to inhibit Vδ2 cells also. This peripheral inhibition of γδT cell subsets could be part of the known systemic immunological adaptation during pregnancy, which could be measured in the peripheral blood also.

A further interesting issue is whether peripheral and decidual γδT cells differ in their NK cell receptor expression patterns. Regarding the proportion of NK receptor-positive cells, decidual Vδ1 or Vδ2 cells do not differ significantly in their inhibitory NK receptor expression pattern from their peripheral counterpart; however, decidual and peripheral DN γδT cells are phenotypically different and therefore show presumably distinct regulation by the non-classical HLA molecules. Here, in contrast to the peripheral DN γδT cells, the binding of the HLA-E molecule can result in both inhibitory and activating signals in the decidua. Whereas HLA-G primarily inhibits decidual DN γδT cells via KIR2DL4 rather than ILT2.

Although activated decidual γδT cells produce angiogenic factors (G-CSF, FGF-2), the presence of HLA-G did not affect their production *in vitro*. G-CSF is also secreted by decidual NK cells and promotes the disorganization of vascular muscles. This, in turn, aids the invasion of the extravillous trophoblast into the spiral arteries, increasing the blood supply in the placental bed (59, 60). FGF-2, on the other hand, activates MAPK signaling and was assumed to improve endometrial receptivity (61). Furthermore, it was demonstrated that FGF-2 improves proliferation and survival of trophoblast organoid cultures *in vitro* (62). Although the concentrations of FGF-2 in our experiments were significantly lower than in the the trophoblast culture experiments, it demonstrates that decidual γδT cells contribute to the creation of a nursing environment for the invading trophoblast. Leptin is produced by trophoblast tissue and is known to support trophoblast invasion (63). Administration of leptin increases HLA-G expression on the EVT *in vitro* (64). In this context, we hypothesize that higher leptin concentrations could be connected to the expression of HLA-G itself, indicating an interdependent relationship between HLA-G expression and leptin secretion. In our experiments, leptin concentrations increased only in the presence of decidual but also peripheral blood γδT cells, without a significant difference between the two experimental settings. This generally emphasizes the relevance of immune cells for leptin secretion. Other angiogenic factors like endoglin, placenta growth factor, or vascular endothelial growth factor, which were detected in our experiments, are known to be secreted by trophoblast tissue (65).

Regarding potential defense mechanisms, decidual γδT cells’ intracellular perforin level correlates positively with NKG2C and ILT2 expression on all γδT subsets and with NKG2A for Vδ1 and DN subsets. However, our experiments do not show altered secretion of perforin in the presence of HLA-E or -G. While innate lymphoid cells utilize various activating and inhibiting receptors for their activity, γδT cells can use their TCR for antigen recognition. Our results suggest no immediate consequences for the isolated presence of either HLA-E or -G. However, long-term consequences are possible and likely. In peripheral blood NK cells, these receptors are not just associated with immediate cell reaction but also with a process of activation threshold alteration called NK cell education (66). We suggest a similar process might also be possible for γδT cells.

Decidual γδT cells secrete high levels of IFN-γ and impressive levels of granulysin. This emphasizes their importance in pathogen defense, as granulysin allows pathogen eradication from virus-infected trophoblast cells without harming the trophoblast itself (67).

Our findings highlight the multifaceted functions and interactions of γδT cells in decidua during the first trimester, confirming the concept that γδT cells are potential effector immune cells at the feto-maternal interface, contributing to healthy pregnancy. The presented data provide further evidence that decidual γδT lymphocytes significantly differ from peripheral γδT cells - they produce angiogenic and immunomodulatory proteins, have conserved or even increased cytotoxic potential, and they could be controlled by non-classical HLA molecules. Accordingly, HLA-G and HLA-E, expressed by EVT can influence decidual γδT cell function through receptors like NKG2C, NKG2A, ILT2, and KIR2DL4, which interactions may modulate immune responses, adding another layer of complexity to the maternal-fetal interface. However, the detailed consequences of the cross-linking of the non-classical HLA molecules and their receptors on the different γδT cell subpopulations remain to be elucidated in the future. Moreover, our study also reveals the potential research interest of the under-researched DN γδT cell population, which could be a promising target for further investigations in reproductive immunology.

## 4 Materials and methods

### 4.1 Human samples

Decidual tissue samples and matched peripheral blood were obtained from healthy pregnant women (n = 27, age *(mean ± SD)* = 25.9 ± 1.4) undertaking an elective pregnancy termination during the first trimester (gestational age *(mean ± SD)*= 9.3 ± 0.3) in the Department of Obstetrics and Gynecology, University of Pécs, Medical School, Hungary.

### 4.2 Isolation of decidual mononuclear cells

The pregnancy was terminated by vacuum aspiration, and the collected tissue was immediately processed. First, the collected decidual pieces were macroscopically homogenized with scissors (approximately 2 mm^3^). Hereafter, the tissue was resuspended with prewarmed (37°C) collagenase type IV (1 mg/mL, Gibco^®^) and transferred to C-tubes (Miltenyi Biotec). To create a single-cell solution, the samples were then further dissected using a gentleMACS™ dissociator (Miltenyi Biotec) with three fast contrarotating cycles (800 rpm/25 sec/cycle) and slow agitation (40 rpm) for one hour at 37°C. After that, the cells were collected through successive 100 µm, 70 µm, and 40 µm nylon cell strainers (Miltenyi Biotec) and washed in RPMI1640 medium (Lonza) supplemented with penicillin (1 x 10^5^ U/L) (Lonza) and streptomycin (0.05 g/L) (Lonza). In the next step, decidual mononuclear cells (DMCs) were isolated by Ficoll-Paque™ (GE Healthcare) gradient centrifugation. The collected cells were washed and resuspended in RPMI1640 medium (Lonza) containing 20% fetal calf serum (Gibco^®^) supplemented with penicillin (1 x 10^5^ U/L) (Lonza) and streptomycin (0.05 g/L) (Lonza). The resuspended cells were distributed onto cell culture dishes and incubated overnight at 37°C and 5% CO_2_ to allow the remaining decidual stroma cells to settle and adhere. The next morning, the non-adherent cells were aspirated, washed, controlled for viability with trypan blue, and split for cryopreservation and isolation of γδT cells. Cryopreserved DMCs were used for flow cytometric measurements, while isolated decidual γδT cells were co-cultured with choriocarcinoma cell lines or soluble HLA proteins.

### 4.3 Isolation of peripheral blood mononuclear cells

Heparinized peripheral blood was diluted with phosphate-buffered saline (PBS), and peripheral blood mononuclear cells (PBMCs) were isolated by Ficoll-Paque™ (GE Healthcare) gradient centrifugation. Hereafter, the collected cells were washed and resuspended in RPMI1640 medium (Lonza) containing 20% fetal calf serum (Gibco^®^) supplemented with penicillin (1 x 10^5^ U/L) (Lonza) and streptomycin (0.05 g/L) (Lonza), then incubated overnight at 37°C and 5% CO_2_. The next morning cells were controlled for viability and split cryopreservation and isolation of γδT cells. Cryopreserved PBMCs were used for flow cytometric measurements, while isolated peripheral γδT cells were co-cultured with choriocarcinoma cell lines or soluble HLA proteins.

### 4.4 Cryopreservation

The washed cells were resuspended in heat-inactivated human serum containing 10% dimethyl sulfoxide and frozen at -80°C utilizing MrFrosty™ Freezing Container (Thermo Scientific™) for later analysis.

### 4.5 Isolation of γδT cells

Decidual and peripheral blood γδT cells were isolated using the ‘TCRγ/δ^+^ T cell Isolation Kit’ (Miltenyi Biotech) according to the manufacturer’s instructions. The purity of the yielded γδT cells was determined by flow cytometry, and samples showing more than 90% γδT cells (γδTCR^+^/all living cells) were used for cell co-culture experiments (n=8). Although the precise composition of the antibody cocktail used in this kit is not publicly available, in-house testing confirmed the efficient elimination of CD56^+^ cells.

### 4.6 Flow cytometry

Matched decidual and peripheral blood cryopreserved cells of 27 participants were thawed and transferred into prewarmed (37°C) RPMI1640 medium (Lonza) supplemented with 10% fetal calf serum (Gibco^®^), penicillin (1 x 10^5^ U/L) (Lonza) streptomycin (0.05 g/L) (Lonza), and DNase (20µg/mL) (Sigma). Then, the cells were washed at 400x*g* for 7 min, resuspended in protein-free PBS, distributed into round-bottom polystyrene tubes (2 x 10^6^/tube), and washed at 400x*g* for 7 min in protein-free PBS. Consecutively, the cells were stained for viability (according to the manufacturer’s instructions) and surface antigens (30 min at RT in the dark). Then, the cells were fixed and permeabilized for intracellular target (Perforin, KIR2DL4) staining utilizing the InsideStain Kit (according to the manufacturer’s instructions) (Miltenyi Biotec). The fluorochrome-conjugated antibodies used in each panel are summarized in **Supplementary Table 1**. Finally, the cells were resuspended in PBS with 1% paraformaldehyde and stored in the dark at 4°C until measurement on a Navios™ flow cytometer (Beckman Coulter). Due to low live cell count or poor sample quality, five decidual and four peripheral blood samples were excluded during preanalytical quality control in FlowJo™. Compensation matrices were calculated by FlowJo™ using CompBeads (BD™) and MACS^®^ Comp Bead Kit, anti-REA (Miltenyi Biotec) for fluorochrome-labeled antibodies, and PBMCs for the viability dye. Gamma/delta T cells were defined as lymphocytes → single cells → ZombieNIR^–^CD45^+^TCRγδ^+^ events. Decidual cells were further defined as residency marker (CD69) positive to exclude peripheral blood-derived cells in the decidual sample (68). All gates are based on fluorescence-minus-one controls (FMO; also see **Supplementary Information 5**). Due to day-to-day variability and the different fluorophores, we standardized fluorescence intensity data to the individual FMO:


$$Standardized\;Fluorescence\;Intensity=\frac{Median_{Population}-Median_{FMO}}{robust\;standard\;deviation_{FMO}}$$

### 4.7 Cell (co-)culture

Three human choriocarcinoma cell lines (JAr) were used as model tissues: A standard JAr cell line (HLA class I^–^) and the two JAr lines transfected with either HLA-E or HLA-G_1m_ (JArE and JArG_1m_, respectively).

The cell lines were donated by P. Le Bouteiller (INSERM UMR 1043, Toulouse, France). JArG_1m_ was produced by transfection of the pCDNA3/*HLA-G1m* plasmid, a gift of Dr. M. Lopez-Botet (Department of Immunology, University Hospital la Princesa, Madrid, Spain), in which the HLA-G leader sequence was modified as follows: the methionyl residue at position 2 was mutated to threonine; therefore, it could not provide a functional signal peptide for the expression of HLA-E ensuring the exclusive expression of HLA-G (69). JAR-E was transfected with a cd3.14 cosmid encoding HLA-E, a gift of M. Ulbrecht (Institute of Anthropology and Human Genetics, Munich, Germany) (70), in which the HLA-E leader sequence was replaced by that of HLA-A2, providing stable peptides for the expression of HLA-E, as described by Lee et al. (71). Upon arrival, aliquots of all cell lines were stored in our liquid nitrogen biobank. Low passage-count aliquots were thawed for our experiments. The cell lines were cultured in RPMI1640 medium (Lonza) supplemented with penicillin (1 x 10^5^ U/L) (Lonza) and streptomycin (0.05 g/L) (Lonza), pyruvate (100mM) (Gibco^®^), geneticin (300 mg/mL) (Gibco^®^) for all transfectants and 10% fetal calf serum (Gibco^®^) at 37°C and 5% CO_2_. The expression of HLA-E or HLA-G was regularly confirmed via flow cytometry (**Figure 3**).

On the day of sample acquisition, cells of all three cell lines were seeded onto 96-well plates (30,000 cells/well). After that, cells were incubated at 37°C and 5% CO_2_ overnight for confluent growth.

On the next day, the old culture medium was carefully aspirated. Then, 100 µL of freshly isolated, matched decidual and peripheral blood γδT cells (10^6^ γδT cells/mL), resuspended in cell line culture medium, were pipetted into the wells. In addition to membrane-bound HLA-E and G_1m_, decidual and peripheral blood γδT cells were incubated with soluble HLA-E (0.5 µg/mL) or HLA-G (0.5 µg/mL) (both from OriGene Technologies) in independent wells. All tests were performed as biological duplicates. All wells were activated using ionomycin (1 µg/mL) (Sigma-Aldrich) and phorbol myristate acetate (25 ng/mL) (Sigma-Aldrich) for 18h. Hereafter, the 96-well plates were centrifuged, and aliquots of the supernatants were cryopreserved at -80°C for batched analysis.

### 4.8 Measurement of cell-(co-)culture supernatants

Diluted (1:10) cell culture supernatants were analyzed for IL-2 (6.5 – 20,000), IL-4 (9.04 – 14,000), IL-10 (5.06 – 14,000), IL-6 (11.58 – 15,000), IL-17A (8.51 – 18,000), TNF-α (12.68 – 12,000), sFas (4.53 – 81,000), sFasL (7.37 – 11,000), IFN-γ (57.13 – 20,000), granzyme A (62.96 – 15,000), granzyme B (24.99 – 52,000), perforin (60.18 – 12,000) and granulysin (175.95 – 57,000) utilizing the Human LegendPlex™ CD8/NK Panel (BioLegend) on a Canto 2 flow cytometer (BD Bioscience) according to the manufacturer’s instructions.

Undiluted cell culture supernatants were analyzed for Angiopoietin-2 (13.7 – 10,000), BMP-9 (2.7 – 2,000), EGF (2.7 – 2,000), Endoglin (13.7 – 20,000), Endothelin-1 (2.7 – 2,000), FGF-1 (13.7 – 10,000), FGF-2 (13.7 – 10,000), Follistatin (27.4 – 10,000), G-CSF (13.7 – 10,000), HB-EGF (1.4 – 1,000), HGF (27.4 – 20,000), IL-8 (1.4 – 1,000), Leptin (137.2 – 100,000), PLGF (6.9 – 1,000), VEGF-A (13.7 – 10,000), VEGF-C (6.9 – 5,000) and VEGF-D (6.9 – 5,000) utilizing the MILLIPLEX^®^ Human Angiogenesis/Growth Factor Magnetic Bead Panel - Cancer Multiplex Assay Enzyme-linked Immunosorbent Assay (Millipore) according to the manufacturer’s instructions. The parentheses’ numbers indicate each cytokine’s detection range (pg/mL).

### 4.9 Statistics and data presentation

All statistical tests were performed in GraphPad Prism 9. Datasets were checked for Gaussian distribution by the D’Agostino-Pearson omnibus normality test. The test used in each comparison is indicated in the respective figure legend. Generally, p-values ≤ 0.05 were considered significant. Illustrations were produced using BioRender and Adobe Illustrator 23.0.4. Plots of flow cytometric data were exported from FlowJo™. Diagrams and Heatmaps were created using GraphPad Prism 9.

## Data availability statement

The original contributions presented in the study are included in the article/**Supplementary Material**. Further inquiries can be directed to the corresponding authors.

## Ethics statement

The studies involving humans were approved by University of Pecs - Medical School, Ethics Committee (5643-PTE 2019, 5643-PTE 2023). The studies were conducted in accordance with the local legislation and institutional requirements. The participants provided their written informed consent to participate in this study.

## Author contributions

JN: Conceptualization, Data curation, Formal analysis, Investigation, Methodology, Project administration, Software, Validation, Visualization, Writing – original draft, Writing – review & editing. PV: Data curation, Investigation, Project administration, Writing – review & editing. IB: Data curation, Investigation, Methodology, Writing – review & editing. BF: Data curation, Formal analysis, Methodology, Validation, Writing – review & editing. AN: Conceptualization, Data curation, Methodology, Validation, Writing – review & editing. ÁB: Data curation, Investigation, Validation, Writing – review & editing. DS: Data curation, Formal analysis, Investigation, Methodology, Writing – review & editing. SE-B: Data curation, Investigation, Methodology, Writing – review & editing. PJ: Data curation, Methodology, Resources, Supervision, Validation, Writing – review & editing. KK: Investigation, Resources, Supervision, Writing – review & editing. ÉM: Writing – review & editing. TB: Funding acquisition, Resources, Supervision, Validation, Writing – review & editing. EM: Validation, Writing – review & editing, Funding acquisition, Project administration, Resources, Supervision. AB: Conceptualization, Data curation, Funding acquisition, Investigation, Project administration, Resources, Supervision, Validation, Writing – review & editing.
