# Supplementary material for: Decidual γδT cells of early human pregnancy produce angiogenic and immunomodulatory proteins while also possessing cytotoxic potential
Source: Front Immunol. 2024 Mar 27;15:1382424. doi: 10.3389/fimmu.2024.1382424 (PMC11004470; doi:10.3389/fimmu.2024.1382424)
Supplement: Supplementary file 1 [file DataSheet_1.docx]

**
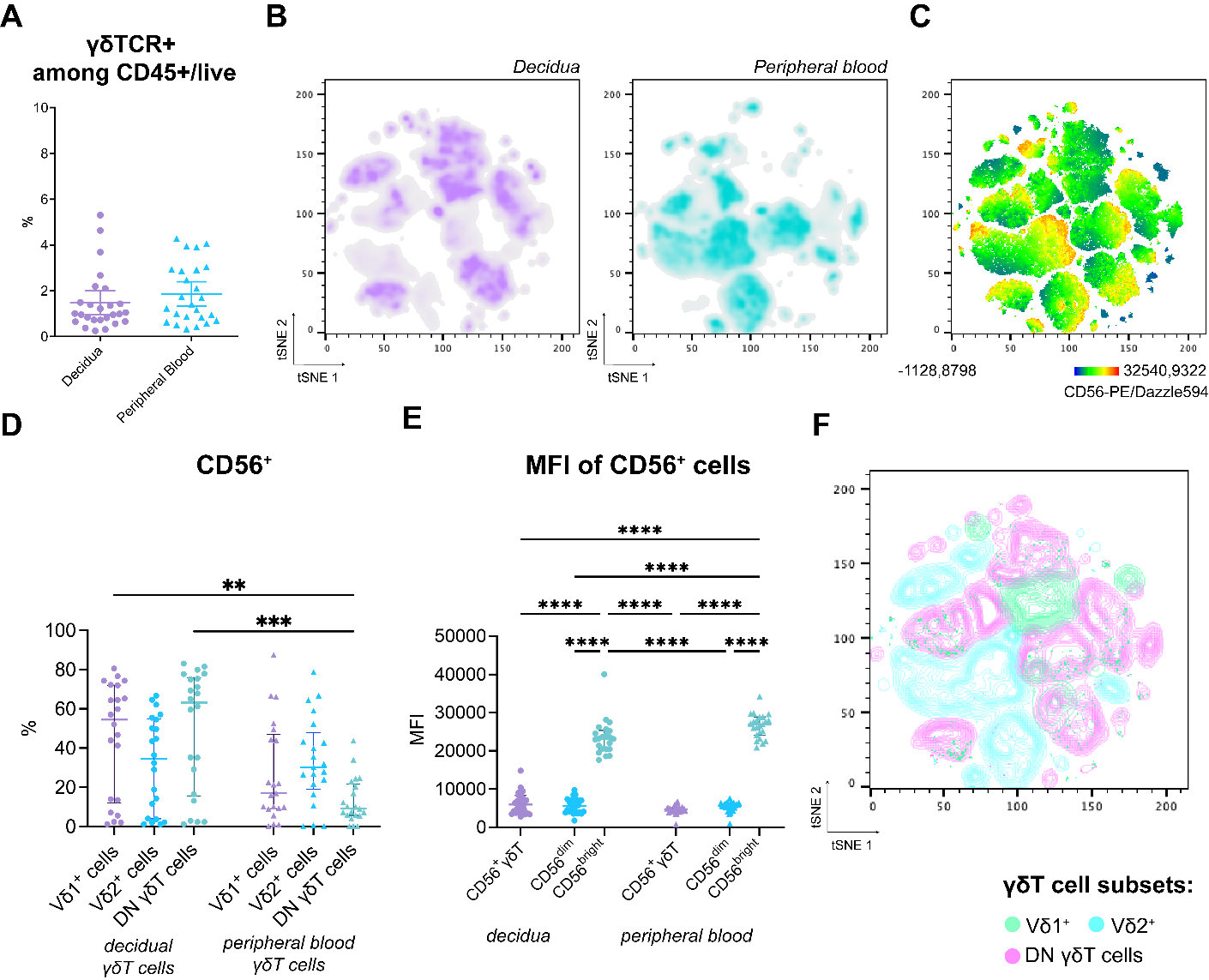
**

**Supplementary Information SI1. Gamma/delta T cells at the early feto-maternal interface:** (A) Statistical comparison of γδTCR^+^ cells' prevalence among CD45^+^/live cells from the decidua (n=22) and peripheral blood (n=23). (B) Isolated depiction of decidual (left) and maternal peripheral blood (right) γδT cells as tSNE density plots (n=1; Panel B). (C) Fluorescence intensity of CD56-PE/Dazzle594™ on clustered γδT cells from peripheral blood and decidua (n=1; Panel B). (D) Statistical comparison of CD56^+^ cells' prevalence among γδT cell subsets from the decidua (n=22) and peripheral blood (n=23). (E) Statistical comparison of CD56-PE-Dazzle™594 Median Fluorescence Intensity (MFI) of CD56+ decidual (n=22) and peripheral blood (n=23) γδT and NK cell populations (F) Contour tSNE plot overlay of Vδ1^+^, Vδ2^+^, and DN γδT cells on γδT cells from peripheral blood and decidua (n=1; Panel B). Testing for significance was performed by the Kruskal-Wallis test with Dunn’s multiple comparisons post-hoc test. **: p ≤ 0.01, ***: p ≤ 0.001, ****: p ≤ 0.0001.


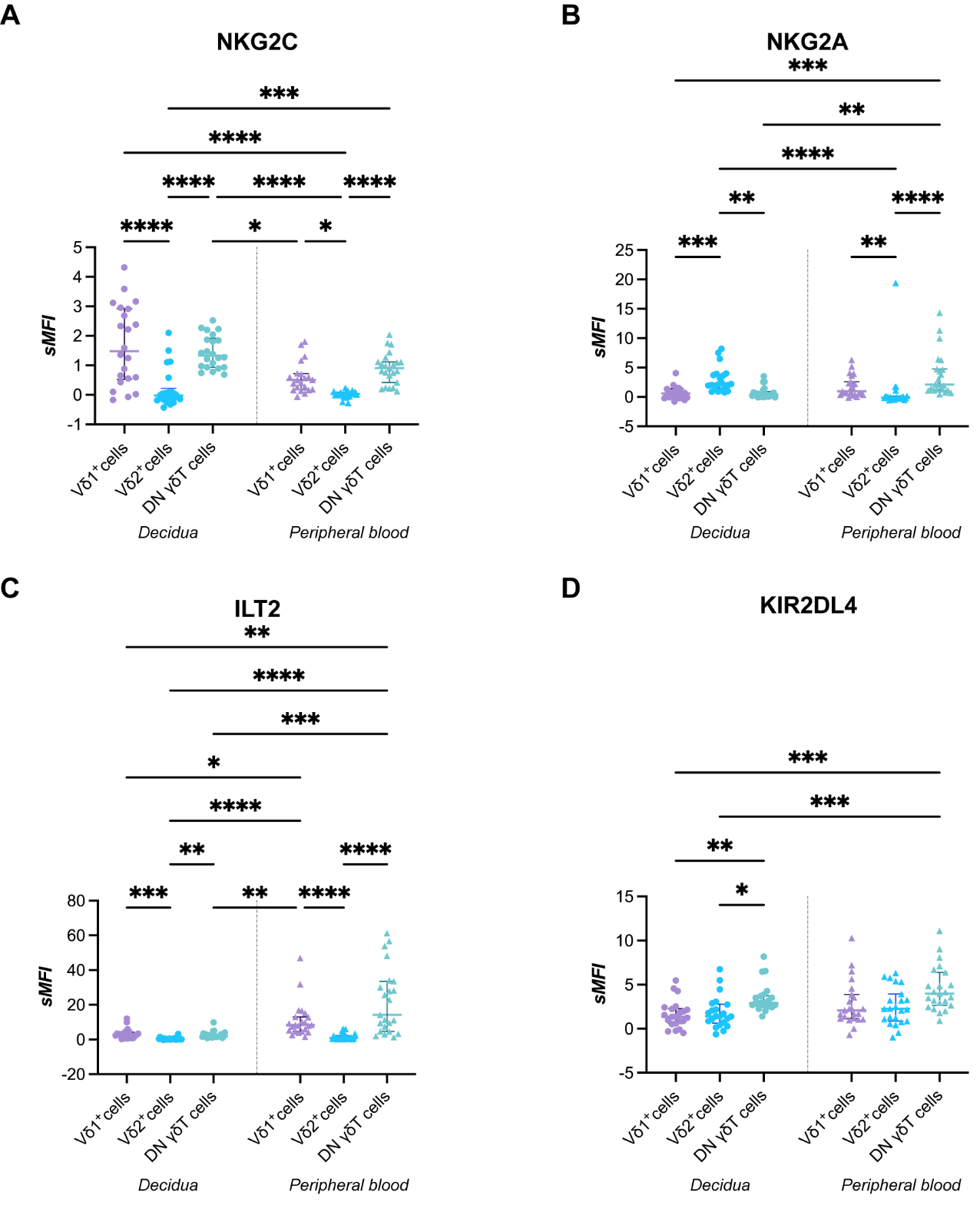


**Supplementary Information SI2. Expression of HLA-E or HLA-G-binding receptors on γδT cells at the early feto-maternal interface:** Statistical comparison of standardized median fluorescence intensity (sMFI=[Median_Subset_-Median_FMO_]/rSD_FMO_) of (A) NKG2C-PE, (B) NKG2A-APC, (C) ILT2-PE, (D) KIR2DL4-APC on decidual (n=22) and peripheral blood γδT cell subsets (n=23). Testing for significance was performed by the Kruskal-Wallis test with Dunn’s multiple comparisons post-hoc test. *: p ≤ 0.05, **: p ≤ 0.01, ***: p ≤ 0.001, ****: p ≤ 0.0001.

**
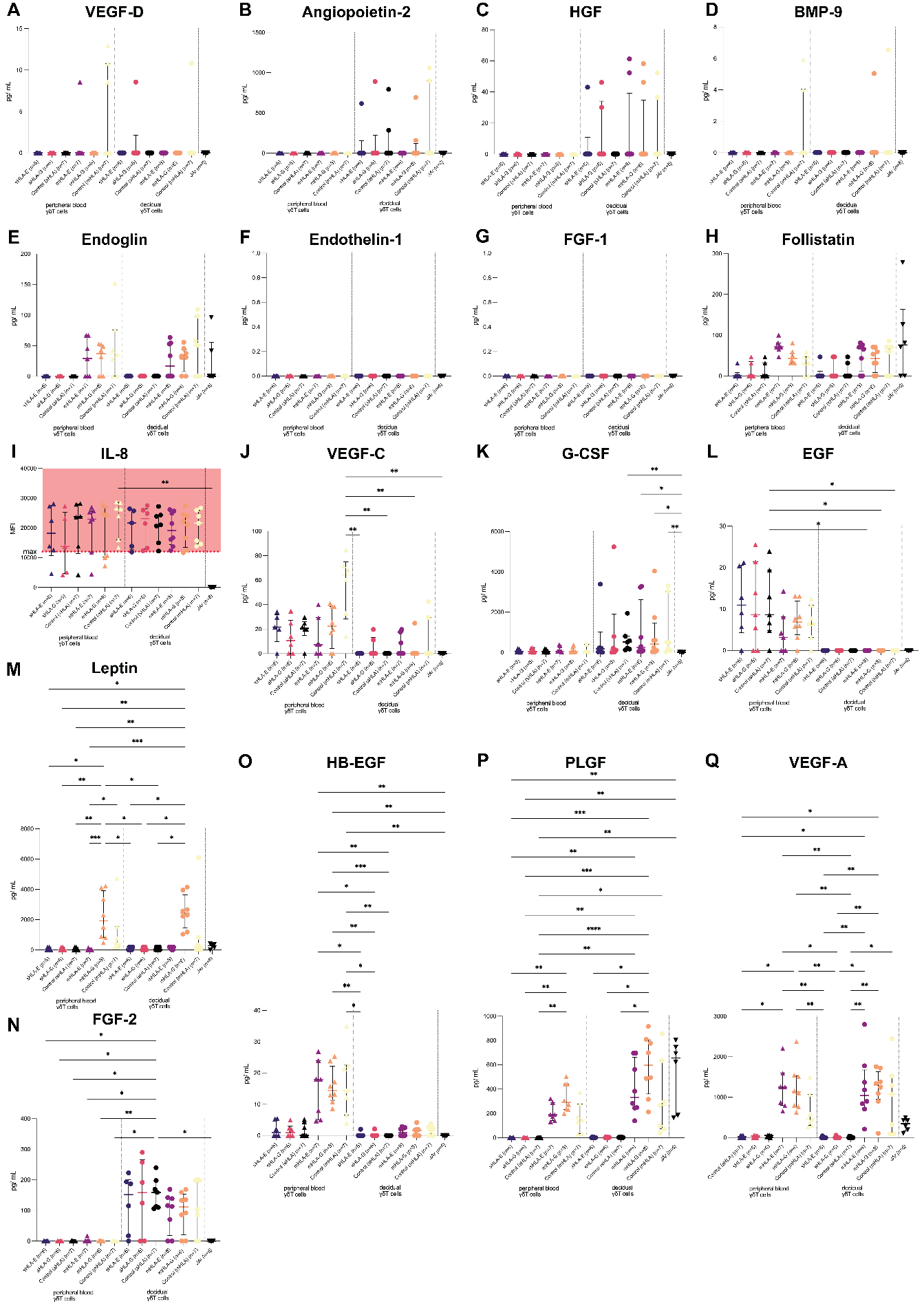
**

**Supplementary Information SI3. Angiogenic factor secretion profile of early decidual γδT cells:** Statistical comparison of measured cytokine concentrations of (A) VEGF-D, (B) Angiopoietin-2, (C) HGF, (D) BMP-9, (E) Endoglin, (F) Endothelin-1, (G) FGF-1, (H) Follistatin, (I) IL-8, (J) VEGF-C, (K) G-CSF, (L) EGF, (M) Leptin, (N) FGF-2, (O) HB-EGF, (P) PLGF, and (Q) VEGF-A in all experimental settings. All statistical comparisons were performed by the Kruskal-Wallis test with Dunn’s multiple comparisons post-hoc test. *: p ≤ 0.05, **: p ≤ 0.01, ***: p ≤ 0.001.

**
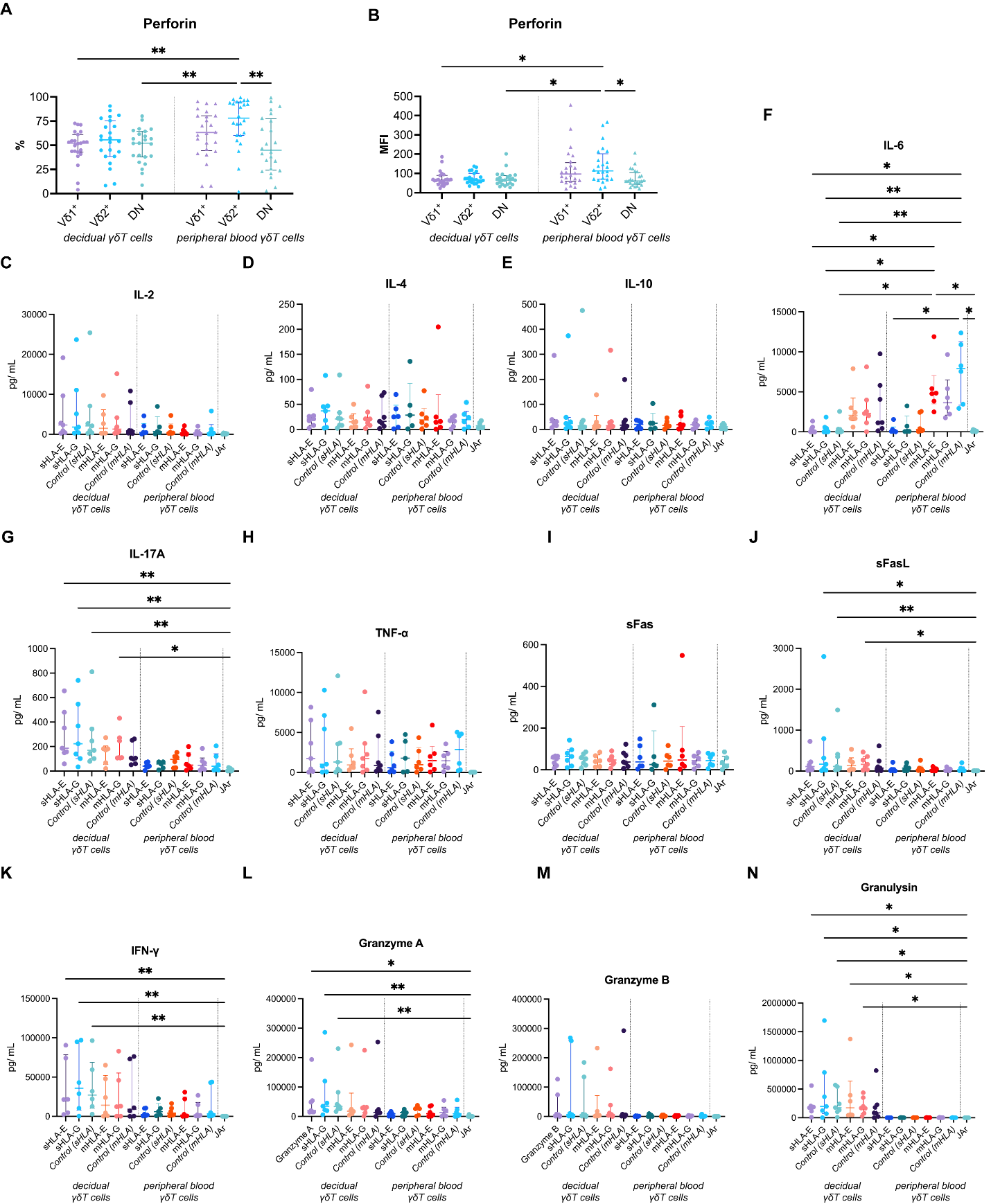
**

**Supplementary Information SI4. Cytotoxic potential of early decidual γδT cells:** (A) Prevalence of Perforin-VioBlue®^+^ cells among all γδT subpopulations of decidual (n=22) and peripheral blood (n=23) samples. Median fluorescence intensity (MFI) of Perforin-VioBlue® of all γδT subpopulations of decidual (n=22) and peripheral blood (n=23) samples. Statistical comparison of measured cytokine concentrations of (C) IL-2, (D) IL-4, (E) IL-10, (F) IL-6, (G) IL-17A, (H) TNF-α, (I) sFas, (J) sFasL, (K) IFN-γ, (L) Granyzme A, (M) Granzyme B, (N) Granulysin in all experimental settings. All statistical comparisons were performed by the Kruskal-Wallis test with Dunn’s multiple comparisons post-hoc test. *: p ≤ 0.05, **: p ≤ 0.01.

**
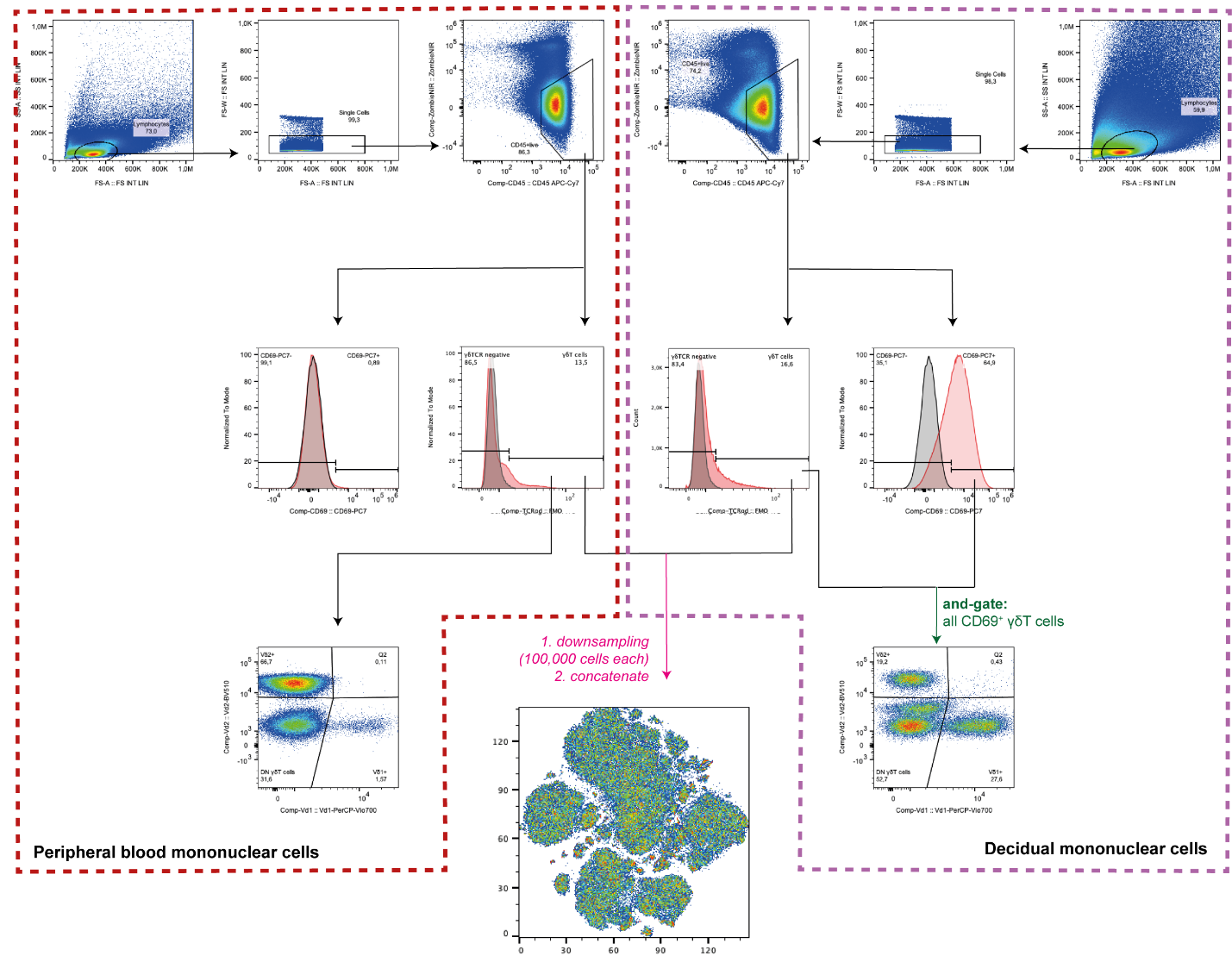
**

**Supplementary Information Figure SI5. Gating strategy for peripheral blood and decidual mononuclear cells:** Lymphocytes (FS-A/SS-A) → Single cells (FS-A/FS-W) → CD45^+^live (CD45-APC/Cy7/ZombieNIR) → γδT cells (TCRγδ-FITC). For dimension reduction, matched γδT cells population from the decidua and the peripheral blood were adjusted to the exact cell count utilizing the DownSampleV3 FlowJo plugin, then concatenated. Hereafter dimension reduction was performed with FlowJo's built-in tSNE algorithm. Gates are based on fluorescence-minus-one (FMO) controls. FMOs are depicted in the single-parameter histograms as a black line. Tissue-resident decidual γδT cells were defined by a Boolean "and"-gate (CD69^+^ and γδT cells).

**Supplementary Table SI6. Flow cytometry panels.**

| **Antibody/ Dye** | **Fluorophore** | **Dilution** | **Clone** | **Host Species** | **Company** | **Cat. #** |
| --- | --- | --- | --- | --- | --- | --- |
| **Panel A** | | | | | | |
| Anti-human-Perforin | VioBlue® | 1:50 | delta G9 | Mouse | Miltenyi Biotec | 130-096-569 |
| Anti-human-Vδ2 | Brilliant Violet 510™ | 1:100 | B6 | Mouse | SONY | 2257160 |
| Anti-human-TCRγδ | FITC (Fluorescein isothiocyanate) | 1:50 | B1 | Mouse | SONY | 2256040 |
| Anti-human-NKG2C | PE (Phycoerythrin) | 1:200 | S19005E | Mouse | SONY | 2475020 |
| Anti-human-CD56 | PE-Dazzle™594 | 1:200 | HCD56 | Mouse | SONY | 2191740 |
| Anti-human-Vδ1 | PerCP-Vio700 | 1:200 | REA173 | recombinant Human | Miltenyi Biotec | 130-120-581 |
| Anti-human-CD69 | PE-Cy7 | 1:200 | FN50 | Mouse | SONY | 2154560 |
| Anti-human-NKG2A | APC (Allophycocyanin) | 1:200 | S19004C | Mouse | SONY | 2475540 |
| Zombie NIR™ | Zombie NIR™ | 1:2000 | N/A | N/A | BioLegend | 423105 |
| Anti-human-CD45 | APC-Cy7 | 1:200 | 2D1 | Mouse | SONY | 2442580 |
| **Panel B** | | | | | | |
| Anti-human-Perforin | VioBlue® | 1:50 | delta G9 | Mouse | Miltenyi Biotec | 130-096-569 |
| Anti-human-Vδ2 | Brilliant Violet 510™ | 1:100 | B6 | Mouse | SONY | 2257160 |
| Anti-human-TCRγδ | FITC (Fluorescein isothiocyanate) | 1:50 | B1 | Mouse | SONY | 2256040 |
| Anti-human-ILT2 | PE (Phycoerythrin) | 1:50 | GHI/75 | Mouse | SONY | 2268540 |
| Anti-human-CD56 | PE-Dazzle™594 | 1:200 | HCD56 | Mouse | SONY | 2191740 |
| Anti-human-Vδ1 | PerCP-Vio700 | 1:200 | REA173 | recombinant Human | Miltenyi Biotec | 130-120-581 |
| Anti-human-CD69 | PE-Cy7 | 1:200 | FN50 | Mouse | SONY | 2154560 |
| Anti-human-KIR2DL4 | APC (Allophycocyanin) | 1:50 | REA768 | recombinant Human | Miltenyi Biotec | 2335040 |
| Zombie NIR™ | Zombie NIR™ | 1:2000 | N/A | N/A | BioLegend | 423105 |
| Anti-human-CD45 | APC-Cy7 | 1:200 | 2D1 | Mouse | SONY | 2442580 |
